# Morphology and thickness of corneal endothelial cells in young Sudanese individuals with myopia

**DOI:** 10.25122/jml-2023-0251

**Published:** 2023-12

**Authors:** Raghda Faisal Mutwaly, Saif Hassan Alrasheed, Abd Elaziz Mohamed Elmadina, Sulaiman Aldakhil

**Affiliations:** 1 Department of Optometry, College of Applied Medical Sciences, Qassim University, Buraydah, Saudi Arabia; 2 Department of Ophthalmic Medical Photography, Faculty of Optometry and Visual Sciences, Al-Neelain University, Khartoum, Sudan; 3 Department of Binocular Vision, Faculty of Optometry and Visual Sciences, Al-Neelain University, Khartoum, Sudan

**Keywords:** endothelial cell density, hexagonality, central corneal thickness, specular biomicroscope, corneal guttata

## Abstract

Deviations in corneal endothelium morphology and thickness may indicate corneal abnormalities and could be associated with myopia development. This study aimed to evaluate corneal endothelial cell morphology and central corneal thickness in young individuals with myopia. A prospective study was conducted at Al-Neelain University Eye Hospital between January 2019 and January 2020, including 160 patients with myopia (320 eyes). Data was gathered through clinical assessment of visual acuity, refractive error, and corneal endothelial cells. Results showed that 60% of participants with myopia were female, with a mean age of 21.99±2.8 years and a mean equivalent sphere of -3.19±2.67D. There was a significant difference in endothelial cell degeneration between myopia groups (P<0.001). Corneal guttata occurred in 9.1% of eyes with low myopia and 68.2% with moderate myopia, whereas polymegathism and polymorphism were more prevalent in high myopia. The mean central corneal thickness was 500.50±38.94 µm in low myopia, 497.02±36.23 µm in moderate myopia, and 477.87±43.625 µm in high myopia (P=0.007). The mean endothelial cell number in low myopia was 107.86±21.12, 106.0±24.03 in moderate myopia, and 101.23±18.49 in high myopia (P<0.05). The mean difference in endothelial cell density, coefficient of variation, and hexagonality in low, moderate, and high myopia was not significant (P>0.05). However, Pearson’s correlation revealed a significant negative correlation between the degree of myopia and central corneal thickness (r= -0.174, P=0.002) as well as endothelial cell number (r= -0.124, P=0.026). The study concluded that central corneal thickness and endothelial cell number significantly decreased with an increase in the degree of myopia. Corneal guttata was the most common form of endothelial cell degeneration observed in cases of high myopia.

## INTRODUCTION

The corneal endothelial layer in humans is the posterior surface of the cornea and consists of cells with a hexagonal shape arranged in a mosaic pattern as a monolayer [1, 2]. The morphology of the cornea is typically characterized using three key metrics: endothelial cell density (ECD), which is the number of corneal endothelial cells per square millimeter (mm^2^); coefficient of variation (COV), calculated by dividing the average cell area by the standard deviation of the cell area; and the proportion of cells that are hexagonally shaped [1, 3, 4]. Endothelial cell density is responsible for regulating the movement of fluid and ions between the aqueous humor and corneal stroma, which is essential for maintaining corneal thickness and transparency and results in good vision [1, 5, 6]. Myopia is a form of refractive error in which parallel rays of light come to a focus in front of the retina when the accommodation is relaxed [7]. High myopia is commonly associated with an increased risk of posterior change, such as cataracts, posterior vitreous detachment, retinal detachment, and myopic macular degeneration [8]. Additionally, it has been reported that both corneal endothelial cells and central corneal thickness (CCT) are impacted in patients with myopia, particularly in cases where myopia exceeds -6.0D [9, 10].

Research has indicated that in eyes with high myopia, the endothelial cell layer of the cornea often exhibits reduced ECD and hexagonality compared to eyes with moderate or low myopia [4]. However, the CCT showed no notable difference between low and moderate myopic eyes. The proportion of hexagonal cells did not significantly vary between high and moderate myopic eyes. Another study indicated that high myopia was associated with abnormal values of COV and hexagonality [11]. Furthermore, a prior study observed significant alterations in the morphology of corneal endothelial cells in moderately myopic Chinese children aged 8 to 9 years [1]. On the other hand, a study reported that the mean spherical equivalent was significantly associated with the changes in corneal curvature and ECD. However, CCT and the COV of cell volume were in the normal range and were poorly correlated with respect to changes in mean spherical equivalent (MSE) [12]. Thomas *et al*. [13] observed that in cases of high myopia, the elongation of the eyeball leads to a decrease in both central corneal thickness and mean endothelial cell density. They suggested that these factors should be assessed before performing anterior segment surgeries and demonstrated a statistically significant difference in the mean corneal endothelial cell count between emmetropic individuals (2812.80 cells/mm^2^) and axial myopes (2653 cells/mm^2^) (P<0.05) [14]. However, the mean central corneal thickness measurements did not show a statistically significant change between emmetrope (490.05 microns) and axial myope (489.37 microns). An earlier study [15] showed that in eyes with greater myopia, the corneal endothelial cell layer tends to have lower endothelial cell density and hexagonality compared to eyes with lower levels of myopia. However, there was no significant difference in the coefficient of variation between eyes with low and moderate myopia. A recent study by Hairol *et al*. [16] reported that corneal curvature correlated significantly with axial length (P=0.001) but not with myopia magnitude (P=0.91). They concluded that myopia magnitude and axial length are more sensitive to detecting changes in corneal curvature in myopes. Despite an impressive body of research, few studies have reported the relationship between corneal endothelium cell characteristics and myopia, and there is still a lack of information on cornea endothelial cell parameters in relation to the degree of myopia. Therefore, the current study was conducted to evaluate corneal endothelium cell morphology and central corneal thickness in young Sudanese individuals with different degrees of myopia.

## MATERIAL AND METHODS

This study was a prospective, cross-sectional, hospital-based research conducted at the Department of Ophthalmic Medical Photography, Al-Neelain University Eye Hospital, from January 2019 to January 2020. We included 320 myopic eyes from 160 individuals diagnosed with myopia. Participants were aged between 16 and 38 years and included both male and female participants.

The inclusion criteria for the study specified patients diagnosed with myopia, having an equivalent sphere (ES) of -1.00 diopters (D) or less, and ocular astigmatism not exceeding -1.00 diopters (D), with myopia correction limited to spectacles only. Participants were required to have normal intraocular pressure (IOP) between 11-20mmHg. Exclusion criteria included a history of contact lens wear, ocular or systemic diseases, a history of refractive surgery, or intraocular surgery.

Data collection involved clinical examinations, including demographic data (age and gender), comprehensive history taking, vision assessment using Decimal notation, refractive error measurement via an auto-ref-keratometer (Topcon RK-2200), and evaluation of corneal endothelial cells morphology using a non-contact specular biomicroscope (Topcon SP-3000). Corneal endothelium assessment included evaluation of CCT, ECD, cell number (CN), COV of cell size, and hexagonality (HEX). All measurements were taken at the central part of the cornea. Participants were classified into three groups according to the degree of myopia: low myopia (>−3.00 to ≤ -1.00D), moderate myopia (>−6.00 to ≤ -3.00D), and high myopia (≥6.00D). Three measurements were taken for each variable for reliability of readings, and then the average was obtained.

Data was analyzed using IBM Statistical Package for Social Sciences (SPSS, v.25). Descriptive statistics (frequencies, minimum, maximum, mean, and standard deviation) were obtained. The mean of study variables (CCT, ECD, CN, COV, and HEX) across all myopia groups were statistically examined for correlations at a 95% confidence level (CL). Moreover, one-way analysis of variance (ANOVA) was used to determine the mean difference between variables. Statistical significance was set at P<0.05.

## RESULTS

The study included 160 patients with myopia (320 eyes), of whom 60 % were women and 40% were men. Their age ranged from 16 to 38, with a mean age of 21.9±2.8 years. Uncorrected VA ranged from .01 to 0.9 (.36±.29), while the best corrected VA ranged from 0.1 to 1 with a mean of .97±.11 showing significant improvement with optical correction (t = -45.70, P>0.001). Refractive correction in sphere equivalent (SE) ranged from –1.00 to – 17.00 D (- 3.19±2.67 D), as shown in Table 1. Furthermore, the one-way ANOVA test revealed significant differences between age and degrees of myopia (F=3.954, P=0.009). Regarding the distribution of myopia, 205 eyes (64.10%) had low myopia (range -1.00 to -2.75, mean: 1.75±0.66D), 84 (26.25%) had moderate myopia (range -3.00 to -5.75, mean: 4.32±0.90D) and 31 (9.69%) had high myopia (range -600 to -17.00, mean: 9.61±3.20D) as shown in Table 2.

**Table 1:** Demographic profile of participants

Parameter	All eyes (n=230)	Male participants (n=128)	Female participants (n=192)		P
**Age (yrs.)**	21.99±2.8	22.94±3.18	21.36±2.39	F=3.954	0.009
**SE (D)**	3.19±2.67	3.20±2.57	3.18±2.74		
**UCVA**	0.33±0.26	0.33±0.27	0.39±0.31	T=-45.70	<0.001
**BCVA**	0.97±0.11	0.97±0.12	0.97±0.11		
**CCT (µm)**	497.40±39.2	501.30±38.62	494.80±39.40	R=-0.174^**^	0.002
**CN**	106.73±21.7	107.91±20.45	105.94±22.54	R=-0.124^*^	0.026
**COV (%)**	32.87±5.8	33.51±5.39	32.43±5.97	R=0.065	0.246
**ECD (mm^2^)**	2837.32±262.3	2834.37±215.54	2839.29±289.85	R=-0.108	0.053
**HEX (%)**	58.26±10.2	57.81±10.37	58.56±10.05	R=-0.013	0.822

SE = sphere equivalent, UCVA = uncorrected visual acuity, BCVA = best corrected visual acuity, CCT = central corneal thickness, CN = cell number, COV = coefficient of variance, ECD = endothelial cell density, HEX = hexagonality.

**Table 2:** Distribution of corneal endothelium parameters among all myopic groups

Corneal parameter	Low (n=205) (>−3.00 to ≤ -1.00)		Moderate (n=84) (>−6.00 to ≤ -3.00)		High (n= 31) (≤−6.00)		P value
	Range	Mean ± SD	Range	Mean ± SD	Range	Mean ± SD	
**SE (D)**	-1.00 to -2.75	1.75±0.66	-3.00 to –5.75	4.32±0.90	-6.00 to -17.00	9.61±3.20	<0.001
**CCT (µm)**	258–608	500.50±38.94	418–561	497.02±36.23	373–579	477.87±43.63	0.007*
**CN**	41–156	107.86±21.12	36–108	106.0±24.03	41–129	101.23±18.49	>0.05
**COV (%)**	22.90–64.70	32.60±5.17	19.80–54.40	33.59±6.71	21.10–53.70	32.67±6.68	>0.05
**ECD (cell/mm^2^)**	2004.9–3780.7	2849.2±269.0	2061.5–3321.4	2834.4±257.3	2447.7–3222.9	2766.35±223.75	>0.05
**HEX (%)**	28–89	58.13±9.74	30–79	58.18±11.15	34–77	59.387±10.40	>0.05

SE = sphere equivalent, CCT = central corneal thickness, CN = cell number, COV = coefficient of variance, ECD = endothelial cell density, HEX = hexagonality.

Considering all eyes, CCT ranged from 258 to 608µm with mean: 497.40±39.2 µm, CN ranged from 36 to 158 (mean: 106.73±21.7), ECD ranged from 2004.90 to 3780.70 (mean: 2837.32±262.3cells/mm^2^), COV ranged from 19.80 to 64.70 (mean: 32.87±5.8 %), and hexagonality ranged from 28 to 89 (mean 58.26±10.2%) as shown in Table 1.

Pearson’s correlation between the degree of myopia and all corneal endothelial cell parameters revealed a significant negative correlation between the degree of myopia and CCT (r= -0.174, P=0.002) as well as CN (r= - 0.124, P=0.026). However, there was no significant correlation between myopia and COV, ECD, and HEX (P>0.05). Moreover, an independent sample test showed no statistically significant differences in CCT, CN, CV, CD, and hexagonality between male and female participants (P>0.05).

In addition, the mean CCT significantly differed across myopia groups: 500.50±38.94 µm in low myopia, 497.02±36.23 µm in moderate myopia, and 477.87±43.625 µm in high myopia (P=0.007). The mean CN was 107.86±21.12 in low myopia, 106.0±24.03 in moderate myopia, and 101.23±18.49 in high myopia, with no significant difference (P>0.05). The mean ECD was 2849.24±269.03 cells/mm^2^ in low myopia, 2834.43±257.32 cells/mm^2^ in moderate myopia, and 2766.35±223.75 cells/mm^2^ in high myopia, with no significant difference (P>0.05). The mean COV was 32.60±5.17 % in low myopia, 33.59±6.71% in moderate myopia, and 32.67±6.68% in high myopia, although the difference was not significant (P>0.05). The mean HEX was 58.13±9.74 % in low myopia, 58.18±11.15% in moderate myopia, and 59.387±10.40% in high myopia (P>0.05), as shown in Table 2.

CCT, CN, COV, CD, and hexagonality did not show significant variation in relation to the degree of myopia (one-way ANOVA, P>0.05). However, a highly significant difference in CCT was observed between low and high degrees of myopia (one-way ANOVA, P=0.007, F=4.604).

**Figure 1:**
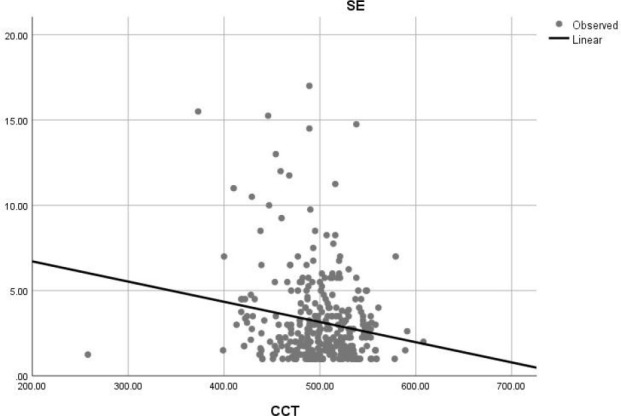
Correlation between the magnitude of myopia and central corneal thickness in the study population

The multivariate regression analysis (F=2.714; P=0.020) showed that only 4 % of the variation in the refractive correction SE was due to CN, COV, ECD, and HEX (R=0.204; R2=0.041). However, this contribution to SE was not significant (P>0.05). The univariate regression showed a statistically significant contribution from CCT (F=9.897; P=0.00; R=0.174, R2=3%), as shown in Figure 1.

The majority of eyes (n=275, 85.9%) had normal corneal endothelial cells, while 22 (6.9 %) eyes had corneal guttata, 16 (5 %) eyes had polymegasthism, and 7 (2.2 %) eyes had polymorphism. In the study, 205 eyes (64.1%) presented a low degree of myopia, 84 eyes (26.3%) had moderate myopia, and 31 eyes (9.7%) were classified as high myopia. There was a significant difference in the occurrence of endothelial cell degeneration among these myopia groups (P<0.001). Specifically, 203 eyes (73.8%) had a normal endothelial cell profile in the low myopia group, while only 2 eyes (9.1%) showed signs of corneal guttata. Among the moderate myopia cases, 62 eyes (22.5%) had a normal endothelial profile, and 15 eyes (68.2%) presented corneal guttata. In the high myopia category, just 10 eyes (3.6%) had a normal endothelium. In contrast, 5 eyes (22.7%) had corneal guttata, 9 eyes (56.3%) showed polymegathism, and 7 eyes (100%) in this group displayed polymorphism, as detailed in Table 3.

Moreover, the chi-square test revealed significant differences between corneal degenerative changes in different myopia groups (P<0.001).

**Table 3:** Distribution of corneal endothelium degenerative changes among all myopia groups

n=320	Low	Moderate	High	Total	P value
Condition	n (%)	n (%)	n (%)	n (%)	
**Normal**	203(73.8)	62(22.5)	10(3.6)	275(85.9)	0.000
**Guttata**	2(9.1)	15(68.2)	5(22.7)	22(6.9)	
**Polymegasthism**	0(0.0)	7(43.8)	9(56.3)	16(5.0)	
**Polymorphism**	0(0.0)	0(0.0)	7(100)	7(2.2)	
**Total**	205(64.1)	84(26.3)	31(9.7)	320(100.0)	

## DISCUSSION

In the current study, there was a significant difference in endothelial cell degeneration between myopia groups, with such changes being more prevalent in high myopia (P<0.001). Corneal guttata was found in low and high myopia, whereas polymegasthism and polymorphism were commonly associated with high myopia. The mean of CCT in myopic groups was thicker in low myopia and thinner in high myopia, and the difference was highly significant (P=0.007). Our findings agree with Chang *et al*. [10], who found that the cornea tended to be thinner in eyes with a high degree of myopia (r = -0.16, P=0.021). However, the thinning of the CCT may be due to the axial elongation of the eye, which is one of the causes of myopia. Conversely, numerous studies [1, 17, 18] have also stated that CCT was not associated with a high degree of myopia. According to the authors' findings, the cornea thinning in myopic eyes did not occur like the sclera. The stretching of the eyeball was probably limited to the peripheral of the eyeball, and it did not significantly impact the CCT.

The present study showed that there was a decreased mean of endothelial cell number in low myopia (107.86±21.12), moderate myopia (106.0±24.03), and high myopia (101.23±18.49), but the difference was not statistically significant (P>0.05). Furthermore, in terms of endothelium cell parameters, the study revealed no significant difference in ECD, CN, HEX and COV in eyes with different degrees of myopia. Conversely, Pearson’s correlation revealed a significant negative correlation between the degree of myopia and CCT (r= -0.174, P=0.002) as well as CN (r= - 0.124, P=0.026). These results disagree with a Chinese study [15], which reported statistically significant differences (P<0.001) in ECD, HEX, CN, and COV in eyes with various degrees of myopia. These may be due to differences in sample size, geographic location, and ethnicity of the study population.

The mean ECD in this study, which included young Sudanese individuals with different degrees of myopia, was 2837.32±262.3 cells/mm^2^. On the other hand, previous population-based studies among Japanese [11] and Pakistani [19] reported slightly lower ECD of 2737±238 cells/mm^2^ and 26547±341 cells/mm^2^, respectively. These variations in ECD might be attributed to age-related factors. Our study focused on young participants, with an average age of 21.99 years, while in the Japanese and Pakistani studies, the mean age was 64.2 and 45.4 years, respectively. Sheng and Bullimore [20] conducted a study on the impact of the degree of myopia on corneal endothelial cells and observed changes in individuals with high myopic refractive power. They reported a significant correlation between high myopia, an increase in the COV, and a decrease in HEX of corneal endothelial cells. Other researchers [10, 21] have suggested that these changes might be due to the corneal endothelium's lack of mitotic activity post-birth. Consequently, endothelial cells are expected to flatten to cover the increased surface area, potentially leading to a decrease. The present study has some limitations. One limitation was the recruitment of patients with myopia from only one eye center due to the lack of specular biomicroscopes in other eye hospitals throughout Khartoum state. Additionally, there was a disproportionate representation of subjects with low myopia compared to those with moderate or high myopia, which might lead to potential biases in the results. Future research should focus on longitudinal studies to track the morphological changes in corneal endothelial cells as myopia progresses and to understand how these changes relate to ocular biometry. Despite the limitations mentioned earlier, the present study has successfully evaluated the morphology of corneal endothelial cells and the central corneal thickness in young Sudanese individuals with different degrees of myopia.

## CONCLUSION

Central corneal thickness was thinner in eyes with high myopia than those with low myopia. Furthermore, there was a significant decrease in central corneal thickness and endothelial cell number as myopia increased. However, the degree of myopia did not affect factors such as corneal endothelial cell density, the coefficient of variation, and hexagonality. Corneal guttata was a common form of endothelial cell degeneration in cases of high myopia.
